# Survey on Cardiologists’ Perspectives on Cardiac Point of Care Ultrasound (POCUS)

**DOI:** 10.24908/pocus.v9i2.17258

**Published:** 2024-11-15

**Authors:** Linda Liu, Christine Chow, Cooper Kersey, Brandon Wiley, Jonathan R Lindner, Andrew M Pattock, Carlos L Alviar, Sula Mazimba, Yoonsik Cho, Kavita Khaira, James N Kirkpatrick, Younghoon Kwon

**Affiliations:** 1 Division of Cardiology, Department of Medicine, University of Chicago Chicago, IL USA; 2 Department of Medicine, University of Washington Seattle, WA USA; 3 Division of Cardiology, Department of Medicine, University of Washington Seattle, WA USA; 4 Keck School of Medicine, University of Southern California Los Angeles, CA USA; 5 Los Angeles General Medical Center Los Angeles, CA USA; 6 Oregon Health Science University Portland, OR USA; 7 Division Cardiovascular Medicine, University of Virginia Medical Center Charlottesville, VA USA; 8 The Leon H. Charney Division of Cardiovascular Medicine, NYU Langone Medical Center, New York University Grossman School of Medicine New York City, NY USA; 9 Department of Artificial Intelligence, Choongang University Seoul KOR; 10 Veteran Affairs Puget Sound Health Care System Seattle, WA USA

**Keywords:** Point-of-Care Ultrasound, Cardiology, Perspectives, POCUS

## Abstract

**Introduction**: Cardiac point of care ultrasound (POCUS) has been used with increasing frequency. As a result of this trend, this study sought to characterize cardiologists’ perspectives on cardiac POCUS. **Methods:** An 18-question survey on demographics, cardiac POCUS clinical practice, education, and infrastructure was distributed by 16 academic medical centers. Likert scale responses were categorized into three groups: 1) “strongly agree” or “agree” 2) “strongly disagree” and “disagree” and 3) “neutral.” **Results:** Of the 140 survey responses collected from January to September 2021, 41% of respondents used cardiac POCUS more than twice in an inpatient week. Seventy-one percent of cardiologists believed that cardiac POCUS should be integrated more regularly into clinical practice and into cardiology fellowship education. Less than half of respondents (44%) reported easy access to POCUS devices, and more than half of respondents (56%) did not think there was appropriate institutional infrastructure to easily upload and document cardiac POCUS images (56%). **Conclusions**: Academic cardiologists had varying opinions on the use and impact of cardiac POCUS. However, most cardiologists believed that cardiac POCUS should be more incorporated within practice despite persisting infrastructure barriers.

## Introduction

The clinical application of cardiac ultrasound revolutionized the practice of cardiology 1. While formal echocardiography is an important component of cardiology practice, the advent of cardiac point of care ultrasound (POCUS) has broadened the use of cardiac ultrasound in the clinic and the hospital 2. Cardiac POCUS is defined as cardiovascular ultrasound that is used at the bedside by a clinician to answer a specific clinical question 3. Unlike a formal echocardiogram, cardiac POCUS does not require a credentialed sonographer, interpretation by a trained cardiologist, structured reports, archived images, or a formal testing facility. Our aim was to characterize the perspectives of cardiologists on cardiac POCUS clinical practice and implementation barriers.

## Methods

An 18-question survey (Table 1) on demographics, cardiac POCUS clinical practice, education, and infrastructure was developed and distributed to echocardiography laboratory directors at 24 major academic medical centers. These institutional contacts then distributed the survey to their cardiology faculty. Likert scale responses were trichotomized, with responses of “strongly agree” or “agree” classified into one group, “strongly disagree” and “disagree” classified into another, and “neutral” responses as the third group. Of the 24 institutions approached, 16 distributed the survey. This yielded 140 survey responses from January 2021 to September 2021. Seventy percent of cardiologists formally interpreted echocardiograms at their institutions.

**Table 1:** Response rates for the survey questions.

**Question**						**n (%)**
**Do you primarily work at an academic medical institution?**						
Yes						135 (96.4)
No						5 (3.6)
**Do you work as an attending in an inpatient setting for at least a portion of the year (e.g.** **wards, consult service, CCU)?**						
Yes						136 (97.1)
No						4 (2.9)
**How many years since fellowship have you been practicing as a cardiologist?**						
Yes						67 (48.2)
No						72 (51.8)
**Do you formally interpret echocardiograms at your institution?**						
Yes						97 (69.8)
No						42 (30.2)
**In a typical week on an inpatient service, how frequently do you use cardiac POCUS?**						
Yes						82 (59)
No						57 (41)
**On average, how frequently do you use cardiac POCUS in the outpatient clinic setting?**						
Yes						121 (91)
No						12 (9)

**Table 0:** 

**Likert Questions**	**Strongly Disagree** n (%)	**Disagree** n (%)	**Neutral** n (%)	**Agree** n (%)	**Strongly Agree** n (%)	**Total N**
I feel confident in my hands-on skills to acquire quality cardiac POCUS images	9 (6.5)	14 (10.1)	20 (14.5)	42 (30.4)	53 (38.4)	138
I primarily rely on my trainees or advanced practice providers (APPs) to scan and acquire cardiac POCUS images	36 (26.3)	36 (26.3)	26 (19)	25 (18.2)	14 (10.2)	137
Use of cardiac POCUS often changes my clinical management	10 (7.2)	18 (13)	38 (27.5)	52 (37.7)	20 (14.5)	138
Cardiologists should incorporate more cardiac POCUS in their practice	4 (2.9)	8 (5.8)	27 (19.7)	57 (41.6)	41 (29.9)	137
I am concerned that increased use of cardiac POCUS will decrease the volume of formal echocardiograms	26 (18.8)	65 (47.1)	28 (20.3)	15 (10.9)	4 (2.9)	138
Cardiac POCUS is time consuming and labor intensive	13 (9.7)	60 (44.8)	35 (26.1)	22 (16.4)	4 (3)	134
Where I practice, POCUS machines are easily accessible	10 (7.2)	33 (23.9)	34 (24.6)	43 (31.2)	18 (13)	138
Where I practice, there is an infrastructure to easily upload and document cardiac POCUS images	26 (19)	51 (37.2)	33 (24.1)	23 (16.8)	4 (2.9)	137
Cardiology fellowship should include more formal education on POCUS in general	6 (4.3)	9 (6.5)	25 (18.1)	67 (48.6)	31 (22.5)	138
Where I practice, Cardiology is often involved in cardiac POCUS training for other specialties	15 (10.9)	45 (32.6)	41 (29.7)	31 (22.5)	6 (4.3)	138
Use of cardiac POCUS by any specialty should be formally credentialed	8 (5.8)	14 (10.1)	35 (25.4)	46 (33.3)	35 (25.4)	138
I am familiar with the billing process for cardiac POCUS	52 (39.1)	53 (39.8)	10 (7.5)	15 (11.3)	3 (2.3)	133

continued Table 1

## Results

Forty-one percent of respondents reported using cardiac POCUS more than twice a week during an inpatient service week. In the outpatient setting, only 9% reported using cardiac POCUS more than two times per ten clinic sessions. Most respondents felt confident in their ability to acquire quality cardiac POCUS images (69%). Faculty who formally interpreted echocardiograms were more likely to report confidence in their cardiac POCUS acquisition skills (p<0.01). About half of the respondents (52%) agreed that cardiac POCUS often impacts their clinical management. Most respondents believed cardiologists should incorporate more cardiac POCUS in practice (71%) and that cardiac POCUS would not decrease formal echocardiogram orders (66%).

Overall, respondents were in favor of cardiac POCUS education in cardiology fellowship (71%). More than half of respondents (59%) agreed with having a formal credentialing process for any specialty that uses cardiac POCUS. Almost half of respondents agreed that they had easy access to POCUS machines (44%), though 25% were neutral and 31% disagreed. About half of respondents did not believe there was appropriate institutional infrastructure to easily upload and document cardiac POCUS images (56%). There was consensus among respondents on their lack of familiarity with the cardiac POCUS billing process (40% disagreed and 39% strongly disagreed with having familiarity). Respondents who did not formally interpret echocardiography more often reported less familiarity with billing (p<0.05).

## Discussion

**Figure 1:**
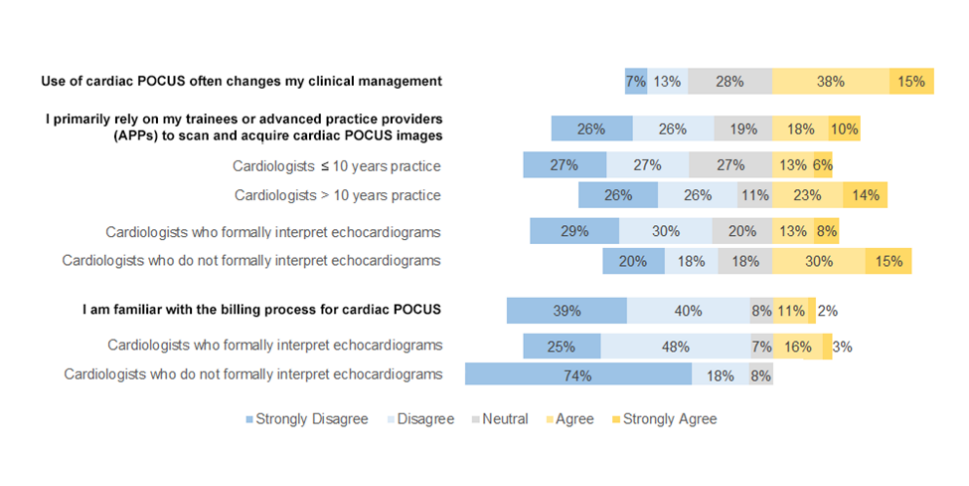
Stacked bar graphs of survey responses regarding (1) cardiac POCUS influence on clinical management (2) who is acquiring cardiac POCUS images, stratified by cardiologists ? 10 years in practice versus > 10 years in practice (p<0.05) and status as a cardiologist that formally interprets echocardiograms vs those who do not (p<0.05) (3) understanding of the cardiac POCUS billing process, stratified by status as a cardiologist that formally interprets echocardiograms vs those who do (p=0.004).

This was a multi-institutional, cross-sectional survey of academic cardiologists’ perspectives on cardiac POCUS clinical practice. We found that cardiac POCUS was frequently employed by cardiologists, more commonly in inpatient settings, and differentially viewed upon in its impact on clinical practice. Cardiologists were generally comfortable with acquiring cardiac POCUS images despite a lack of formalized cardiac POCUS education, likely because of transferable echocardiography experience. However, the current paradigm for echocardiography in the United States exclusively relies on sonographers to acquire images. This is challenging for cardiologists to maintain their hands-on ultrasound skills. Confidence with image acquisition also varied amongst cardiologists, as non-echocardiography faculty were less likely to report confidence with their skills. Our survey suggests that challenges for cardiac POCUS use by cardiologists are related to infrastructure, including access to devices and software for image upload (Figure 1). Addressing the ease of cardiac POCUS at an institutional level by understanding and troubleshooting specific local challenges may improve the practice within cardiology. Additionally, standards for image interpretation, documentation, and archiving, are crucial to ensure adequate quality, adherence to accepted clinical standards, and competence definitions by major societies 4. Reimbursement is another potential issue reflected by our survey; however, our survey question regarding familiarity with the billing process is obscured by the fact that there is no specific procedural billing code for cardiac POCUS. Currently, reimbursement is mostly provided through coding for the performance of a limited transthoracic echocardiogram (TTE). However, billing for a limited TTE requires specific parameters related to image interpretation, documentation and storage 5. Notably, our survey determined a significant difference in billing familiarity between cardiologists who formally interpreted echocardiograms versus those who did not, with the former having greater familiarity with echocardiography billing codes.

In our survey, many cardiologists agreed on the importance of credentialing and training for cardiac POCUS. The American Society of Echocardiography has published standards for training and certification in cardiac POCUS 6, 7. However, establishing a formal pathway and incorporating it in trainees’ schedules are essential for the development of a cardiac POCUS curriculum.

One key limitation in this study is response bias. Those who are more enthusiastic in cardiac POCUS may have been more likely to participate in the survey; furthermore, questionnaires were not distributed by a third of the institutions approached. There may also be variations dependent on subspecialty of respondents. In addition, our survey was distributed only at academic medical centers. Nevertheless, academic centers often set the stage for future practice since they are training the next generation of practitioners. Future directions include a larger survey with a question regarding subspecialty and a potential expansion to non-cardiology providers.

In conclusion, we found varied opinions on the use and clinical impact of cardiac POCUS amongst academic cardiologists. Nonetheless, there was support for incorporating more cardiac POCUS in practice and promoting cardiac POCUS education within cardiology. Infrastructure concerns regarding device accessibility and software for image storage were highlighted as barriers to further adoption of cardiac POCUS.

## Participant Consent

Informed consent was obtained at the time of participant invitation to the survey. An invitation email was sent to potential participants and described the study objectives and anonymous, voluntary nature of the study, indicating that starting the survey would mean consenting to the study. This consent language was also displayed on the survey webpage.

## Disclosure

YK reports support from the NIH (R01HL158765, R21AG070576, R21HL167126). YC reports support from the Institute of Information & communications Technology Planning & Evaluation (IITP) grant funded by the Korea government (MSIT) (No.2021-0-01341, AI Graduate School Program Chung-Ang University). The authors declare that they have no competing interests.

## References

[R252598632149081] Singh S, Goyal A (2007). The origin of echocardiography: a tribute to Inge Edler. Tex Heart Inst J.

[R252598632149079] Pattock A M, Kim M M, Kersey C B (2022). Cardiac point-of-care ultrasound publication trends. Echocardiography.

[R252598632149083] Johri A M, Glass C, Hill B (2023). The Evolution of Cardiovascular Ultrasound: A Review of Cardiac Point-of-Care Ultrasound (POCUS) Across Specialties. Am J Med.

[R252598632149080] Walter J M, Satterwhite L, Lyn-Kew K E (2020). POINT: Should the Use of Diagnostic Point-of-Care Ultrasound in Patient Care Require Hospital Privileging/Credentialing? Yes.. Chest.

[R252598632149085] Hughes D, Corrado M M, Mynatt I (2020). Billing I-AIM: a novel framework for ultrasound billing. Ultrasound J.

[R252598632149084] Labovitz A J (2020). Introducing ASE's Critical Care Echocardiography (CCE) Specialty Interest Group!. J Am Soc Echocardiogr.

[R252598632149082] Kirkpatrick J N, Grimm R, Johri A M (2020). Recommendations for Echocardiography Laboratories Participating in Cardiac Point of Care Cardiac Ultrasound (POCUS) and Critical Care Echocardiography Training: Report from the American Society of Echocardiography. J Am Soc Echocardiogr.

